# The impact of OTU sequence similarity threshold on diatom‐based bioassessment: A case study of the rivers of Mayotte (France, Indian Ocean)

**DOI:** 10.1002/ece3.4701

**Published:** 2018-12-18

**Authors:** Kálmán Tapolczai, Valentin Vasselon, Agnès Bouchez, Csilla Stenger‐Kovács, Judit Padisák, Frédéric Rimet

**Affiliations:** ^1^ MTA‐PE Limnoecology Research Group Veszprém Hungary; ^2^ UMR CARRTEL INRA Thonon‐les‐Bains France; ^3^ Department of Limnology University of Pannonia Veszprém Hungary

**Keywords:** Diatoms, high‐throughput sequencing, OTU, pollution assessment, sequence similarity threshold, taxonomic resolution, Water Framework Directive

## Abstract

Extensive studies on the taxonomic resolution required for bioassessment purposes have determined that resolution above species level (genus, family) is sufficient for their use as indicators of relevant environmental pressures. The high‐throughput sequencing (HTS) and meta‐barcoding methods now used for bioassessment traditionally employ an arbitrary sequence similarity threshold (SST) around 95% or 97% to cluster sequences into operational taxonomic units, which is considered descriptive of species‐level resolution. In this study, we analyzed the effect of the SST on the resulting diatom‐based ecological quality index, which is based on OTU abundance distribution along a defined environmental gradient, ideally avoiding taxonomic assignments that could result in high rates of unclassified OTUs and biased final values. A total of 90 biofilm samples were collected in 2014 and 2015 from 51 stream sites on Mayotte Island in parallel with measures of relevant physical and chemical parameters. HTS sequencing was performed on the biofilms using the *rbcL* region as the genetic marker and diatom‐specific primers. Hierarchical clustering was used to group sequences into OTUs using 20 experimental SST levels (80%–99%). An OTU‐based quality index (Idx_OTU_) was developed based on a weighted average equation using the abundance profiles of the OTUs. The developed Idx_OTU_ revealed significant correlations between the Idx_OTU_ values and the reference pressure gradient, which reached maximal performance using an SST of 90% (well above species level delimitation). We observed an interesting and important trade‐off with the power to discriminate between sampling sites and index stability that will greatly inform future applications of the index. Taken together, the results from this study detail a thoroughly optimized and validated approach to generating robust, reproducible, and complete indexes that will greatly facilitate effective and efficient environmental monitoring.

## 1 INTRODUCTION

Benthic diatoms are widely used as ecological indicators for bodies of water due to their short generation time, large diversity, high and sensitivity to environmental changes (Mann & Vanormelingen, 2013). They serve as a proxy for the entire phytobenthos (Kelly, King, Jones, Barker, & Jamieson, 2008), which is one of the five biological quality elements (BQEs) required by the European Water Framework Directive (WFD) for the assessment of the ecological quality of bodies of water (European Commission, 2000).

Diatom‐based quality indices are generally calculated using a weighted average equation (Zelinka & Marvan, 1961) based on the species’ ecological optimum and tolerance, as defined by its abundance profile along a pollution gradient. Each index has its own reference database that contains the ecological values (optimum and tolerance) of a set of species. Diatom studies are largely dedicated to rigorously characterizing specimens down to the finest taxonomic level possible (species, subspecies), even though it is often challenging and not necessary for bioassessment purposes (Lavoie, Dillon, & Campeau, 2009; Rimet & Bouchez, 2012). Microscopy‐based identification of diatoms is based on their morphological attributes and thus carries several drawbacks. The process is time‐consuming and requires experienced analysts. Furthermore, misidentifications are common and cause discrepancies in the species inventories of different laboratories, which must be regularly rectified (Kahlert et al., 2009, 2012 ). Moreover, different indices may have different ecological values for the same species because their profiles were defined from different ecoregions with limited range of environmental variables (Besse‐Lototskaya, Verdonschot, Coste, & Vijver, 2011).

DNA barcoding has enabled the rapid development of novel molecular techniques that have greatly improved the quality of species identification (Hebert, Cywinska, Ball, & deWaard, 2003). These approaches employ standard markers to identify taxa‐specific sequences in the DNA of the organisms in question to serve as that organism's barcode. These DNA‐based methods are efficient and reduce misidentifications due to phenotypic plasticity (Leliaert et al., 2014) or cryptic diversity (Kaczmarska, Mather, Luddington, Muise, & Ehrman, 2014). High‐throughput sequencing (HTS) technology, in combination with the aforementioned meta‐barcoding, allows for simultaneously identifying multiple taxa from multiple environmental samples (Taberlet, Coissac, Pompanon, Brochmann, & Willerslev, 2012). This makes the routine analysis of environmental samples faster, more cost‐effective, and accurate than traditional microscopy‐based methods and provides much information than ever before. This facilitates expanding the sampling network to include more sites monitored on a more frequent basis and has thereby revolutionized the field of biomonitoring (Baird & Hajibabaei, 2012; Keck, Vasselon, Tapolczai, Rimet, & Bouchez, 2017). The incorporation of molecular techniques in biomonitoring has caused remarkable progress over the past decade in terms of optimal genetic marker selection (Kermarrec et al., 2013), HTS platform (Loman et al., 2012; Shokralla, Spall, Gibson, & Hajibabaei, 2012), DNA extraction (Vasselon, Domaizon, Rimet, Kahlert, & Bouchez, 2017), and the bioinformatics required to analyze the HTS data.

Sequence data obtained from the HTS platform are subjected to a quality‐filtering process and then typically clustered into operational taxonomic units (OTU). Three main approaches have been developed to effectively cluster sequences into OTUs (Westcott & Schloss, 2015). And which algorithm to apply depends on many factors, including the target taxa, the genetic markers, and the sequencing method (Flynn, Brown, Chain, MacIsaac, & Cristescu, 2015). The closed‐reference clustering method compares sequences to a reference database and then clusters into OTUs based on similarity to the reference sequence. The most commonly used clustering approach is de novo clustering. Here, sequences are clustered into OTUs before taxonomic assignment. Hierarchical clustering is a form of de novo clustering that creates a distance matrix to compute sequence dissimilarity between all sequence pairs before generating the OTUs. While this method is widely used, it requires high computational capacity (Sun et al., 2012). Greedy heuristic clustering is a more computationally effective approach because it does not compare all of the sequence pairs but, instead, analyzes one input sequence at a time. If the distance between that sequence and an already existing OTU is smaller than the predefined threshold, the sequence is assigned to the existing OTU. If not, it serves as the seed sequence for a new OTU (Sun et al., 2012). Both the hierarchical and the greedy heuristic clustering methods use a defined yet arbitrary clustering threshold, called the sequence similarity threshold (SST), as a cutoff value to ensure that the sequences within an OTU are identical (Patin, Kunin, Lidström, & Ashby, 2013). The third approach is termed open‐reference clustering and involves closed‐reference clustering followed by de novo clustering. Thereby, this approach essentially combines the strengths of the two aforementioned methods (Westcott & Schloss, 2015).

While SST values can reach up to 99% (Apothéloz‐Perret‐Gentil et al., 2017), most range between 95% and 97% (Edgar, 2013; Elbrecht & Leese, 2015; Kelly et al., 2018; Patin et al., 2013), which is thought to effectively maximize genetic diversity while also minimizing the frequency of sequencing errors in the resulting HTS‐based dataset (Birtel, Walser, Pichon, Bürgmann, & Matthews, 2015; Schloss & Handelsman, 2005). These thresholds are treated as quasispecies level delimitations regardless of the specific marker, clustering method, or model organism used, even though these parameters can greatly affect the final OTU composition (Flynn et al., 2015).

A taxonomic name is then assigned to each newly generated OTU by comparing a representative sequence, generally the most abundant (Patin et al., 2013), to reference barcodes available in public databases (Rimet et al., 2016). Most studies use a consensus confidence threshold (Schloss et al., 2009) to delineate the abundance of the representative sequences required within an OTU and those that fall below this threshold are labeled as “unclassified OTUs” (Rivera, Vasselon, Jacquet, et al., 2018b; Visco et al., 2015). To generate complete reference libraries, these unclassified OTUs must be resolved (Groendahl, Kahlert, & Fink, 2017; Vasselon, Rimet, Tapolczai, & Bouchez, 2017). This challenge represents a considerable and pressing issue because a portion of the taxonomic diversity of the site remains unknown. As such, the quality index calculation will only be based on the ecological values from a portion of the species or genera while others go unidentified, among which may be dominant, relevant species (Rivera, Vasselon, Jacquet, et al., 2018b). An alternative approach was proposed (Apothéloz‐Perret‐Gentil et al., 2017) that avoids the taxonomic assignment of OTUs by using the ecological values of the OTUs directly.

The aim of this study was to determine the impact of taxonomic resolution on a quality index using molecular data. Toward this end, we carried out DNA‐meta‐barcoding on environmental biofilm samples collected from streams on the main island of the Department of Mayotte, a French archipelago in the Indian Ocean. We then investigate the impact of the SST on the OTU quality index (Idx_OTU_) using the abundance profiles (ecological profiles) of the different OTUs that result from a gradient of different SSTs (80%–99%).

We hypothesize that the SST serves as a proxy for taxonomic resolution whereby OTUs of high SSTs represent fine taxonomic characterization (e.g., species, populations) and OTUs of low SSTs represent coarser taxonomic classification (e.g., genera, families). Our approach is similar to studies that analyzed the effect of taxonomic resolution on classical diatom quality indices (Lavoie et al., 2009; Rimet & Bouchez, 2012).

Additionally, we hypothesize that at low SSTs, the ecological profile of an OTU is the result of merging good indicator sequences and thus results in a low‐performance quality index according to its capacity to separate “high‐” and “poor‐”quality samples from each other. On the other hand, at high SSTs, the ecological profiles of the more rare OTUs are based on fewer data points. Thus, they are more sensitive to the outliers that do not fit in the model provided by the ecological profile. We, therefore, hypothesize that after an “optimal” point, increasing the SST results in a less stable index that does not confer additional benefit in terms of quality evaluation. We suggest that an optimal SST, analogous to the optimal taxonomic resolution in previous studies on microscopy‐based approaches (Rimet & Bouchez, 2012), can be identified that maximizes the index's performance and stability.

The effect of the SST on Idx_OTU_ was analyzed from several aspects: (i) the number of OTUs defined at each SST, (ii) the proportion of OTUs identified at the species and genus level after taxonomic assignment (this aspect was studied but not included in the index development), (iii) the performance of the quality index using three different indicators: (iii‐a) the correlation between quality values and the reference environmental gradient, (iii‐b) the index's capability to discriminate between sites with different quality values, and (iii‐c) the variability in the index values conferred by the process used for index development (stability).

## 2 MATERIALS AND METHODS

### 2.1 Study site and sampling network

**Figure 1:**
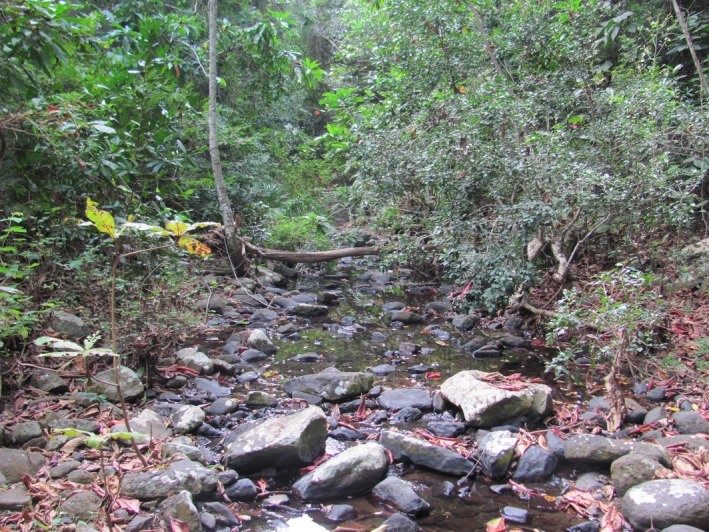
One of the sampling site in Dapani River, Mayotte. Good quality sites are typically characterized by dense riparian vegetation, natural river bank, and low turbidity. Since the sampling was carried out during the dry season, the environmental conditions are stable and the water level is low

The French overseas Department of Mayotte is an island in the Comoros archipelago located in the Indian Ocean, northwest of Madagascar (12°50′35″S 45°08′18″E; Appendix S1). Following the change to its legal status in 2011, the implementation of the Water Framework Directive (WFD) became obligatory for its bodies of water (Figure 1). Toward this end, a surveillance monitoring network (RCS) was set up in 2008. This network was complemented with a “reference” (REF) network in 2013 and a “polluted” (POLL) network in 2014 to enlarge the environmental gradient. The classification of sites into these networks was predefined and based, in general, on visible conditions of the area (Tapolczai, Bouchez, Stenger‐Kovács, Padisák, & Rimet, 2017). For the purpose of this study, a total of 90 samplings were collected from the three monitoring networks: 30, 23, and 37 samples from the RCS, POLL, and REF networks, respectively. These were collected from 51 river sites in 2014 and 2015, in parallel with the physical and chemical data associated with each site (Appendix S2).

### 2.2 Phytobenthos sampling, physical, and chemical parameters

The phytobenthos sampling procedure followed both French and European standards (Afnor, 2014, 2016) and was carried out during the dry season (July–August) when flow conditions are more stable compared those the rainy season, which are affected by heavy flooding. The samples were collected using clean toothbrushes to remove the biofilm from the surface of at least five stones from lotic regions. These were then preserved by adding sufficient 99% ethanol to ensure a final concentration of over 70%. The sampling and analysis of the physical and chemical parameters were carried out during the same time period according to APHA standards (APHA, 2012).

### 2.3 HTS procedure

Total DNA was extracted from 2 ml of each phytobenthos samples using the GenEluteTM‐LPA method. A detailed protocol can be found in previous publications (Chonova et al., 2016; Kermarrec et al., 2013). This method is preferred for diatom meta‐barcoding (Vasselon, Domaizon, et al., 2017) because it uses multiple lysis mechanisms (mechanical, enzymatic, and heat‐based) that when combined greatly increase the efficiency of diatom cell lysis and DNA yield.

A short 312‐bp segment of the *rbcL* gene was used as the DNA marker and amplified by PCR using an equimolar mix of a modified version of the Diat_rbcL_708F forward and the R3 reverse primers (Rimet, Abarca, et al., 2018a; Vasselon, Rimet, et al., 2017). Each DNA sample was amplified in triplicate using 1 µl of extracted DNA in a final reaction volume of 25 µl. Detailed information on the PCR mixture and amplification conditions is summarized in Appendix S3.

The three PCR replicates of each DNA sample were pooled and purified using Agencourt AMPure beads (Beckman Coulter, Brea, CA, USA). The quality and quantity of the purified amplicons were checked using the 2200 TapeStation (Agilent Technologies, Santa Clara, CA, USA). Following the library preparation method described by Vasselon, Domaizon, et al. (2017), individual A‐X tag adapters (Ion ExpressTM Barcode Adapters, Life Technologies, Carlsbad, CA, USA) were ligated to the amplicons using the NEBNext® Fast DNA Library Prep Set for Ion TorrentTM (BioLabs, Ipswich, MA, USA). The sample libraries were pooled into two mixes corresponding to the Mayotte 2014 and 2015 sampling campaigns that contained 49 and 41 samples, respectively. Each mix was adjusted to a final concentration of 100 pm and sequenced independently on two Ion 318TMChip Kit v2 (Life Technologies, Carlsbad, USA) using the PGM Ion Torrent machine.

The sequencing was performed by the “Plateforme Génome Transcriptome” (PGTB, Bordeaux, France) who provided one fastq file per sample for the 90 libraries with demultiplexed DNA reads. A quality‐filtering step excluded DNA reads under 250 bp with a Phred quality score below 23 over a moving window of 25 bp, more than one mismatch in the primer sequence, a homopolymer over 8 bp, or an ambiguous base. The 90 trimmed files were merged in order to manipulate all of the samples concurrently using the bioinformatics processes described in Vasselon, Rimet, et al. (2017) using the Mothur software (Schloss et al., 2009). In addition to bioinformatics, the DNA reads were dereplicated to obtain individual sequence units (ISUs). The abundance of ISUs, corresponding to the number of sequence replicates per ISU, was used to remove ISUs with only one sequence. Retained ISUs were then clustered into OTUs using different SSTs ranging from 80% to 99%. Finally, 20 OTU lists, corresponding to each threshold and including the number of DNA reads within the 90 samples, were produced. Based on the taxonomy assigned to each ISU with the classify.seq command (RDP classifier with bootstrap cutoff = 85%, Wang, Garrity, Tiedje, & Cole, 2007) and the R‐syst::diatom library (Rimet et al., 2016, 13–02–2015: R‐Syst::diatom v3, https://www.rsyst.inra.fr/en), a consensus taxonomy was provided to each OUT using the classify.otu command with a confidence threshold of 80%. The Supplementary Data contains the following: the Fastq files with the demultiplexed DNA reads (Appendix S4); information on the sequencing depth before and after trimming (Appendix S5); the final OTU summary, including the proportion of DNA reads, representative DNA sequences for each OTU, and their taxonomic assignments (Appendix S6); descriptions of the sampling sites (Appendix S7); and the script run in Mothur from trimming to obtain the used OTU lists (Appendix S8).

### 2.4 The development and testing of Idx_OTU_

**Figure 2:**
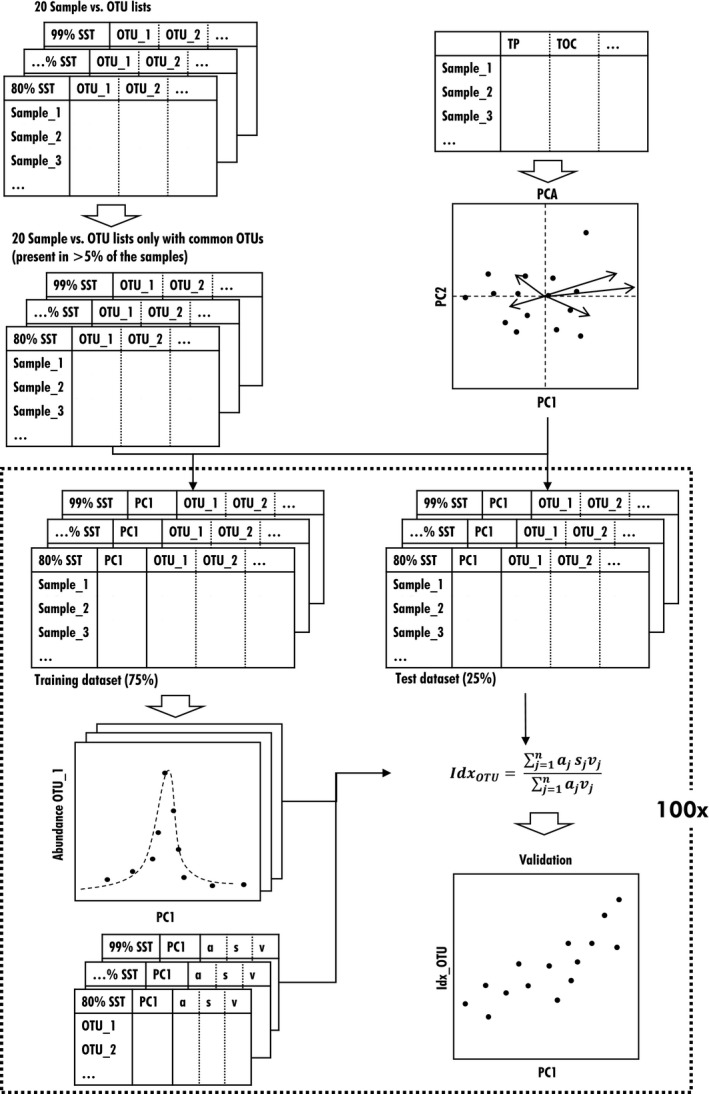
Schematic representation of the index development process and statistical analyses

Sequence reads were transformed into relative abundances in order to normalize the OTU database. Although this is not the ideal approach toward achieving comparable quantification between samples, it is one of the most frequently used, second to rarefying (McMurdie & Holmes, 2014). Additionally, rare OTUs were removed from the 20 OTU lists and only those present in more than the 5% of the samples were kept, resulting in a total of five samples from the original 90 (Figure 2). This arbitrary limit, well established in previous studies (Bere, Mangadze, & Mwedzi, 2014; Stenger‐Kovács, Buczkó, Hajnal, & Padisák, 2007), was necessary to keep a minimum number of samples based on which robust ecological profiles of the OTUs are ensured.

**Figure 3:**
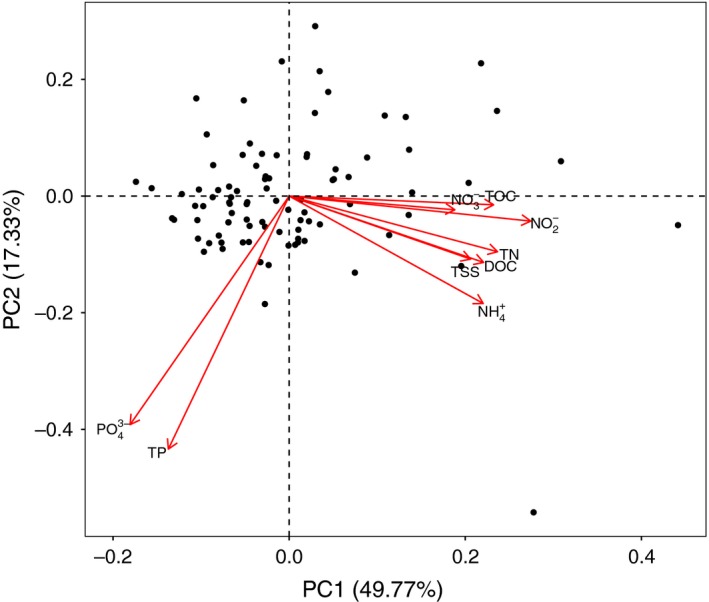
Two‐dimensional graphical representation of principal component analysis results. The environmental variables in this analysis were ammonium (NH_4_^+^), dissolved organic carbon (DOC), nitrate (NO_3_^−^), nitrite (NO_2_^−^), phosphate (PO_4_^3−^), total nitrogen (TN), total organic carbon (TOC), total phosphorus (TP), and total suspended solids (TSS). Sample locations on the primary axis (PC1) represent the reference pressure gradient, from “high” to “poor” quality

Principal component analysis (PCA) was executed using the “prcomp” function in R (R Development Core Team, 2008; Venables & Ripley, 2002) to study the structure of the samples and their relationship to the environmental (physical and chemical) variables (Figures 2, 3). We used the variables shown to be related to anthropogenic pressure in previous study: turbidity [NFU], total suspended solids (TSS [mg/L]), dissolved organic carbon (DOC [mg/L]), total organic carbon (TOC [mg/L]), total nitrogen (TN [mg/L]), total phosphorus (TP [mg/L]), nitrite (NO_2_^−^ [mg/L]), nitrate (NO_3_^−^ [mg/L], phosphate (PO_4_^3‐^ [mg/L]), and ammonium (NH_4_^±^ [mg/L]) (Tapolczai et al., 2017). Logarithmic transformation was applied to the environmental variables in order to ensure the normal distribution required for PCA. The first axis of the PCA (PC1) represents the reference pressure gradient; that is, the position of the samples along this gradient represents the “reference” quality to which the Idx_OTU_ values were compared. The values and summary statistics describing the environmental variables are presented in Appendix S2.

All 20 datasets were randomly divided according to the 20 SST levels into a training dataset containing the 75% of the samples including their position along PC1 and their associated OTU relative abundances and into a test dataset containing the remaining 25% of the samples (Figure 2). Therefore, the index could be tested on an independent dataset (test) that was not included in index development (training). Although the selection of the two datasets was random, the proportions of the samples belonging to the three sampling networks (see Section 2.1) were maintained (0.41, 0.33, and 0.26 for REF, RCS, and POLL, respectively), to ensure a reasonable range of the pressure gradients. At each SST, a random selection of datasets was executed 100 times to measure the average and standard deviation of the Idx_OTU_ values at each sample instead of a single measure that could bias the results. The 100 iterations also allowed for all of the samples to be included in the training and test datasets too. This resulted in 100 training and test datasets at each SST. The whole process resulted in 100 indices tested for each of the 20 SSTs datasets (2,000 indices in total). It is important to note that quality values in the results only contain the Idx_OTU _values calculated on the test dataset.

The ecological profiles of the OTUs in the training datasets were defined by modeling the relative abundance of each OTU in the samples along PC1 (Figure 2). Weighted averages and standard deviations of the profiles were calculated to estimate the ecological optimum (*s*) and the tolerance (*v*) values of the OTUs. The Zelinka‐Marvan equation (Zelinka & Marvan, 1961) was adapted to our data to define Idx_OTU_:

$${\text{Idx}}_{\text{OTU}}=\frac{\sum_{j=1}^{n}a_{j}s_{j}v_{j}}{\sum_{j=1}^{n}a_{j}v_{j}},$$

where *a_j_* = relative abundance of OTU *j*, *s_j_* = sensitivity value or optimum of OTU *j*, and *v_j_* = indicator value or tolerance of OTU *j *in the sample. Sensitivity and indicator values for each OTU were calculated from the abundance values plotted as functions of the samples’ PC1 values. The two ecological values of each OTU comprised a database that was used together with the abundance of the OTUs in the samples for which the index was calculated. Only the data from the training dataset were used to define these profiles. The Idx_OTU _was calculated for each site in the test dataset and then correlated with its position on PC1 (Figure 2).

### 2.5 Idx_OTU_ performance

Three different parameters were examined to assess Idx_OTU_'s performance.

**Figure 4:**
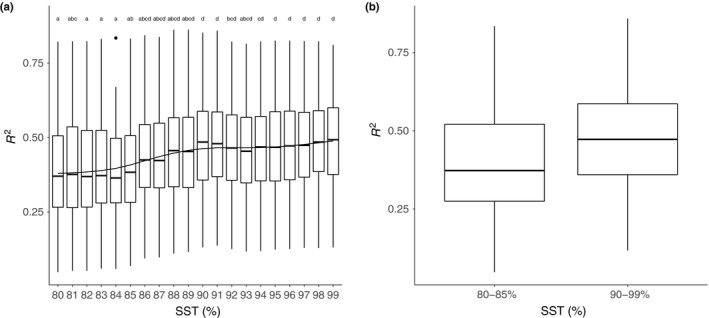
Linear regression coefficients (*R* ^2^) obtained using linear models fitted to the relationship between Idx_OTU_ and PC1 values from the test dataset are presented for each SST (a). The difference in the *R* ^2^ values between the SSTs of 80%–85% (mean = 0.39) and 90%–99% (mean = 0.47) was statistically significant (Student's *t* test, *p* < 0.01).

**Figure 5:**
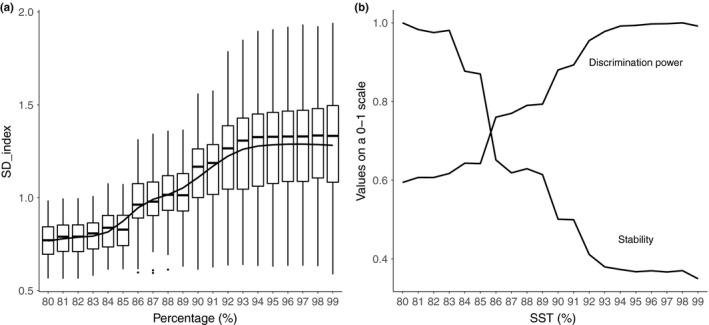
Standard deviations from the Idx_OTU_ values (SD_index) of samples within each of the 100 randomization steps were calculated and plotted against the associated SST (a). The mean (reflective of the discrimination power of the index) and the standard deviation (reflective of the stability of the index) of the SD_index as a function of the SST, normalized to a scale ranging from 0 to 1 (b)

**Figure 6:**
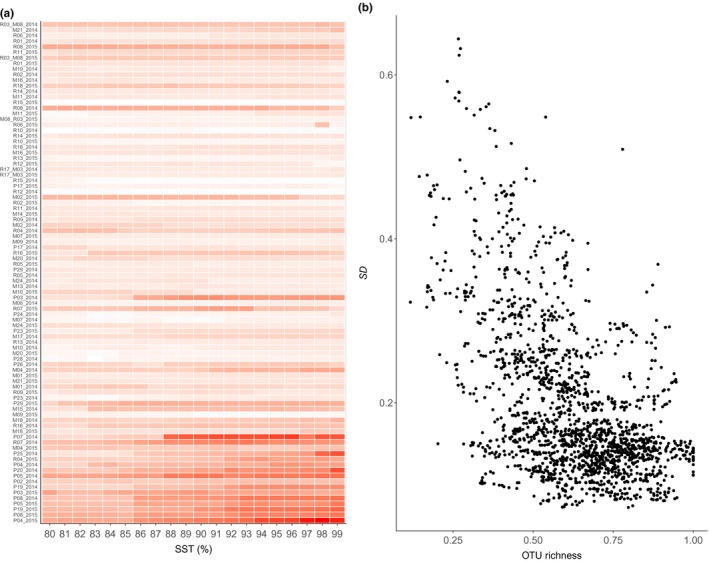
Variability (standard deviation) in the Idx_OTU_ values for each sample as a function of the associated SST. (a) Dark red cells represent higher standard deviation while the lighter colors indicate lower standard deviations. (b) Plot of standard deviations against OTU richness illustrates their statistically significant negative correlation (Pearson's correlation test, *p* < 0.01, *r* = −0.54).

1. Fitting a linear model using the “lm” function in R (Chambers, 1992; R Development Core Team, 2008) to ascertain the relationship between the calculated Idx_OTU_ values and their reference quality conditions (PC1). At each SST level, 100 linear models were fitted due to the 100 randomizations used when selecting the training and test datasets. The regression coefficients (*R* ^2^) of the linear models were used to reflect the performance of the index. These *R* ^2^ values were then plotted as a function of the SST, as described in Section 2.1, 3.2.2 and in Figure 4.
2. Another aspect of the index's performance was studied through the variability of the Idx_OTU_ values among the samples within each randomization step and SST. For this purpose, the standard deviations (*SD*) of the calculated Idx_OTU_ values for the samples within each randomization step were compared and then plotted against the SST gradient (Figure 5). Here, the *SD* was considered as a proxy for the discrimination power of the index, that is, the ability to distinguish between samples with different index values from each other (see Section 3.2.3 and Figure 5).
3. The stability of the index was tested by its ability to reproduce the same results over the course of the 100 randomization steps within each SST. Stability values were calculated for (*a*) the index, using the *SD* of the discrimination powers (see Section 3.2.4 and Figure 4) and for (*b*) the samples themselves, using the *SD* of the Idx_OTU_ values per samples (see Section 3.2.5 and Figure 6).

## 3 RESULTS

### 3.1 Taxonomic resolution and number of OTUs

**Figure 7:**
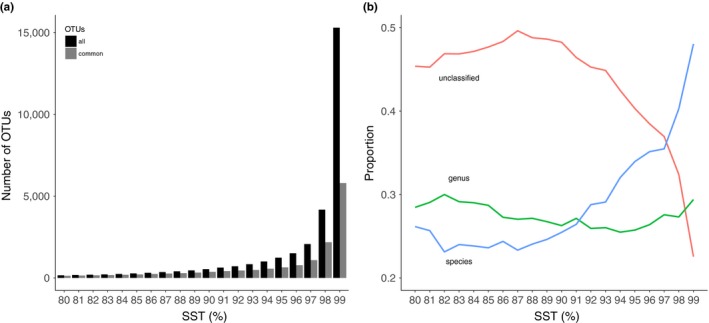
Number of OTUs (a) across the SST gradient before (black) and after (gray) removing the rare ones (present in less than 5% of the samples). Taxonomic affiliation of the OTUs with each experimental SST (b): proportion of OTUs identified at the species and genus level and of unclassified OTUs

Of the 20 OTU lists, the number of OTUs increased exponentially with the SST (Figure 7a). The number of OTUs ranged from 159 at 80% SST to 15,296 at 99% SST. Common OTUs, those that were present in over 5% of the samples, showed similar trends; however, the ratio of the removed rare OTUs increased too: at 99%, over 60% of the OTUs were removed, whereas at 80%, the percentage dropped to only 18%. Assigning taxonomy to the OTUs revealed that the taxonomic resolution changed dramatically with the SST (Figure 7b). From 80% to 93%, the percentage of unclassified OTUs varied between 45% and 50% and then steeply decreased with SSTs over 93% (Figure 7b). The proportion of OTUs identified at the species level exhibited the opposite trend. For SSTs up to 90%, the proportion was around 25%, followed by a sharp increase that reached approximately 50% at 99% SST (Figure 7b). The proportion of OTUs identified at the genus level did not display such a clear pattern, with variations from 25% to 30% across the SST gradient (Figure 7b).

### 3.2 Index performance

#### 3.2.1 Relationship between Idx_OTU_ and the reference gradient

After developing Idx_OTU_ on the training dataset for each SST and randomization step, quality values were calculated for the corresponding test datasets. Then, the relationship between the calculated index values and PC1 was studied using linear models and their *R* ^2^ (Figure 4a). The range of the *R* ^2^ values is always high, due to the outlier datasets generated during the randomization process. The index's performance showed an increase between 85% and 91% SST. This increase was evaluated by comparing the difference in *R* ^2 ^values between the 80%–85% and 91%–99% SSTs (Figure 4b). It increased significantly from a mean value of 0.39 to 0.47 (Student's *t* test, *p* < 0.01). The correlations between the index values were calculated on the samples from the test dataset, and the PC1 values were statistically significant in ~95% of the cases.

#### 3.2.2 Discrimination power of Idx_OTU_

The SDs of the Idx_OTU_ values (SD_index) among the samples from each of the randomization and SST steps were calculated and then used to reflective discrimination power (Figure 5). An increase in the SD_index was observed along the SST gradient with a steep transition at 86%–93% at which point it reached a plateau without any further increases (Figure 5a).

#### 3.2.3 Stability of Idx_OTU_

Concomitantly with the increase in discrimination power, we observed an increase in the interquartile ranges of the boxplots (Figure 5a). The *SD* of the SD_index was used to estimate the stability of the discrimination power against the 100 random selection processes applied to generate the training and test datasets. Higher *SD* values correspond to higher levels of variability in the discrimination power of the 100 randomization steps at one given SST. The observed increase in the discrimination power and associated decrease in stability along the SST gradient is presented in Figure 5b. Values of both parameters were standardized to a scale that ranges from 0 to 1.

#### 3.2.4 Stability of the samples’ Idx_OTU_ values

Figure 6a depicts the variation (also measured in *SD*) in Idx_OTU_ values due to the randomization steps for each sample at each SST. The samples are ordered by mean Idx_OTU_ value on the y‐axis from poor to high quality. This graphical representation illuminates that the quality values of the samples at the two ends of the quality gradient varied greatly. Mainly, the poor‐quality samples had more variation in their Idx_OTU_ values and this variation increased with the SST. The variability in the Idx_OTU_ values of samples in the middle of the pressure gradient was lower, with only a few exceptions (Figure 6a).

#### 3.2.5 OTU richness and index stability

We linked the instability of the Idx_OTU_ values with OTU richness (number of OTUs per samples). The index value variability of each site (*SD*) and OTU richness (number of OTUs normalized across the experimental SSTs) correlated negatively, with higher *SD* values corresponding to low richness and lower *SD* being associated with higher richness (*r* = −0.54, *p* < 0.05; Figure 6b).

## 4 DISCUSSION

### 4.1 Sequence similarity threshold and taxonomic resolution

In the present study, the SST used for clustering sequences into OTUs was regarded as a proxy for taxonomic resolution but because this assumption can be up for debate, it requires further explanation. As discussed comprehensively by Mann (1999), the lack of a solid conceptual basis for diatom taxonomy has resulted in a rapidly changing, unstable classification system of diatoms. Original approaches were based on the morphological characteristics of the specimens but the inclusion of DNA barcoding techniques (Hebert et al., 2003) helped create a taxonomy based on morphology and supplemented by molecular characters. Another important aspect to consider is that both the intragenomic variation and the intraspecies variation of the barcoding gene differ among taxa (Hamsher, Evans, Mann, Poulíčková, & Saunders, 2011). Although we used a hierarchical clustering method with predefined global thresholds in the current study, there are other clustering methods available that we have not tested. The importance of clustering methods presented in the introduction should not be overlooked and warrants further analysis in further studies.

The choice of appropriate taxonomic resolution for bioassessment purposes is a common and active topic of debate in biomonitoring most every biota studied. The identification to the lowest taxonomic level is important for complex ecological questions, fundamental studies, and for simply expanding the common knowledge base. One of the arguments in favor of precise taxonomic resolution (i.e., species‐level) is built on the fact that species represent the basic units of an ecosystem and a clear and thorough understanding of their ecological niches directly impacts the amount, quality, and value of the information they provide (Salmaso, Naselli‐Flores, & Padisák, 2015). However, in practice, classifying specimens to species (or finer) taxonomic level does not necessarily further inform or improve bioassessment. For example, aquatic macroinvertebrates are usually identified down to species or genus level, depending on the taxa and the life stages of the organisms. However, several studies have been unable to find significant differences in the bioindication efficiency of the same community at different taxonomic levels (even family) for the same type of pollution (Bailey, Norris, & Reynoldson, 2001; Bowman & Bailey, 1997). Furthermore, the applicability depends on the metrics being used. For macroinvertebrates, more complex metrics exist than for diatoms, including functionality, life‐forms, and habitat preferences. A comprehensive study by Schmidt‐Kloiber and Nijboer (2004) showed that while some metrics were not sensitive to changes in the taxonomic level (e.g., richness, diversity measures), others (e.g., functional metrics) did impact final quality values.

The question is of greater relevance for the study of protists, where the microscopic identification at the species level is technically challenging and labor intensive (Lavoie et al., 2009; Rimet & Bouchez, 2012). Published studies on the effect of taxonomic resolution are contradictory, and their results seem to depend largely on the index characteristics. For instance, Lavoie et al. (2009) studied the effect of reducing the taxonomic resolution to the genus level for the Eastern Canadian Diatom Index. They found that while it could still successfully separate impacted sites from reference ones, its ability to detect fine changes in the environment had diminished. Other studies, however, have found that the change from species level to genus level does not impact bioassessment efficiency. This has been tested using indicators of river regulation (Growns, 1999) and organic and nutrient pollution (Rimet & Bouchez, 2012).

DNA barcoding has enabled the detection of intraspecific variations that were not detectable using microscopy‐based analysis (Keck et al., 2017; Vasselon, Rimet, et al., 2017). Generally, a sequence similarity of 95% has been used for species‐level delimitation when meta‐barcoding diatoms. In this study, 1,239 OTUs at 95% similarity were identified, which is clearly dwarves the 382 species identified by microscopy (Tapolczai et al., 2017). Such striking differences between the results obtained through microscopy and HTS are commonly reported in the study of diatoms (Rivera, Vasselon, Jacquet, et al., 2018b). These differences are largely due to the cryptic diversity common to diatoms; indeed, it has been shown in several species that the genetic diversity is substantially richer than the morphological diversity (Evans, Wortley, Simpson, Chepurnov, & Mann, 2008; Mann et al., 2004; Souffreau et al., 2013). This relatively newfound ability to recognize these cryptic species is important because their ecological niches may differ even when they live in sympatry (Kelly, Trobajo, Rovira, & Mann, 2015; Rovira, Trobajo, Sato, Ibáñez, & Mann, 2015).

While the effect of taxonomic resolution on bioassessment has been extensively studied using microscopy‐based identification, “OTU studies” have relied on arbitrary clustering thresholds until now. Our study revealed maximum index performance at a 91%–92% SST, lower than traditionally thought necessary. However, it must be noted that the validity and applicability of this threshold are potentially limited to the conditions included in the present analysis (e.g., pollution gradient, community structure) and further experimental consideration and validation are required prior to being exported for widespread use.

### 4.2 Performances of the OTU‐based indices depend on the SST choice

In the present study, we developed a diatom index, based on the same principles as classical ones (e.g., PSI, Coste, 1982; BDI, Coste, Boutry, Tison‐Rosebery, & Delmas, 2009; TDI Kelly, 1998). However, in this case, we directly applied the ecological profile of the OTUs, without taxonomic assignment, avoiding the problem associated with incomplete DNA reference databases, which can easily inject bias (Apothéloz‐Perret‐Gentil et al., 2017; Groendahl et al., 2017; Rivera, Vasselon, Jacquet, et al., 2018b; Zimmermann, Glöckner, Jahn, Enke, & Gemeinholzer, 2015). Even though the number of species included in the DNA reference libraries is constantly increasing (Rivera, Vasselon, Jacquet, et al., 2018b), the proportion of OTUs that can be assigned to the species level remains far from satisfying. Published reports have described a wide range of classification coverage, including 35.7% (Vasselon, Rimet, et al., 2017), 35% (Apothéloz‐Perret‐Gentil et al., 2017), 23% (Rivera, Vasselon, Jacquet, et al., 2018b), and as low as 10% for marine samples (Rivera, Vasselon, Ballorain, et al., 2018a). Our approach is similar to those of Apothéloz‐Perret‐Gentil et al. (2017) but the method for defining the OTUs’ indicator and sensitivity values used in the Zelinka–Marvan equation (1961) is different. In Apothéloz‐Perret‐Gentil et al.’s study, sites were preclassified using the original Swiss morphology‐based index and served as the reference from which the OTU indicator and sensitivity values were defined. In the present study, we directly incorporated the environmental pressure gradients of both physical and chemical parameters using multivariate analysis. Thus, the method described here is completely independent of morphology‐based taxonomy. The disadvantage, however, is that rare OTUs have unreliable ecological profiles and must be removed to safeguard the accuracy and of a system that is most effective when only based on robust OTUs. Our results indicate that the index values calculated for the test dataset correlated significantly with the pressure gradient regardless of the SST; however, an important transitional zone in the SST gradient was observed from 86% to 91%, described by an increasing *R* ^2^ value.

One technical drawback of the index described in this investigation is that when new samplings are carried out in Mayotte, the OTUs generated from these new data may differ from those obtained from the datasets used our index development. Indeed, the sequence composition of the sampling sites can fluctuate over time. This means that the ecological profile, together with the representative sequence of an OTU, must be fixed and assigned to the correct OTU, generated by another sequencing run. However, this requires the calibration, standardization, and extensive validation of the OTU clustering method given its potential to greatly effect final OTU composition.

Interesting, our data uncovered an important trade‐off between the index's discrimination power and its stability. The stability of Idx_OTU _during the randomization process decreased with increasing SSTs. This is due to the exponential increase in OTUs, many of which become less frequent and a higher number warrant removal. Regardless, these OTUs consist of fewer sequences, and thus, their ecological profile cannot be established with any robustness. Thus, the biasing effect of one outlier abundance data point becomes higher and this makes the dataset very sensitive to the random selection process for the training and test datasets. In contrast, coarse taxonomic resolution generates fewer OTUs with wider but more reliable ecological profiles; this leads to a more stable Idx_OTU _with weaker discrimination power. This instability was particularly important when samples presented with low OTU richness. Low OTU richness is observed at highly polluted sites, where only a few resistant species could survive (Blanco et al., 2012; Stevenson, Pan, & Van Dam, 2010) and in reference sites where nutrient limitation has selected for a limited number of taxa (Blanco et al., 2012; Stevenson, Hill, Herlihy, Yuan, & Norton, 2008). A technical drawback of using a high similarity threshold is the elevated risk of misappropriating biological relevance to artifacts and sequence errors, which results in biased and inaccurate results (Patin et al., 2013).

An ecological disadvantage of the de novo hierarchical clustering used in this study is that it hinges on a single, global SST regardless of the species being considered. Given the differential levels of intra and interspecies genetic variation, a clustering method that tailors the SST to the specific characteristics of each taxon would be a valuable tool. Without this, there is always a risk of undergrouping heterogeneous sequences and thereby creating an ecological profile that is not indicative or representative of some taxa, while simultaneously running the risk of overgrouping and thereby separating groups of sequences with similar ecological preferences for other taxa. Further studies should implement approaches similar to that described by Preheim, Perrotta, Martin‐Platero, Gupta, and Alm (2013) that employs the ecological preferences of bacterial sequences (termed distribution‐based clustering) to refine the OTUs.

### 4.3 The ecologically naïve paradigm of diatom indices

The taxonomy‐free approach delineated in this study proposes a solution to overcome the considerable technical challenge posed by incomplete reference databases. However, it is important to highlight that the fundamental basis of Idx_OTU _and the classical taxonomy‐based diatom indices are the same: using uncritically the relative abundance of a list of species (or OTUs) and their ecological values to develop an index calculated from a weighted average equation that correlates with the physical and chemical parameters of an environment. These fundamental aspects have been analyzed in several previous studies: Instead of using this ecologically naïve approach, other groups have proposed reconsidering the functional aspects underlying the ecological indication, for example, using relative biovolume instead of relative abundance (Tapolczai et al., 2017), trait‐based functional groups (B‐Béres et al., 2016), or diversity metrics (Blanco et al., 2012). Nevertheless, the indices currently employed in the EU WFD are criticized for their lack of ecological basis (Schneider, Hilt, Vermaat, & Kelly, 2016) and all of the indices based on molecular techniques use the same naïve approaches (Kelly et al., 2018; Leese et al., 2016), which are qualitatively not ideal. In this study, we are clearly not advocating the uncritical widespread use of Idx_OTU _but instead used it as a test object to assess the effect of the SST was tested.

Sequencing methods can potentially address some of these drawbacks. It has been shown in a previous study that DNA read abundances, using the *rbcL* marker correlate reliably with species’ relative biovolume (Vasselon et al., 2018), thus enabling the generation of more ecologically relevant taxa quantification data (Tapolczai, 2017). Moreover, molecular methods facilitate the analysis of other benthic taxa beyond diatoms (e.g., Cyanobacteria, Chlorophyta, Rhodophyta) without the need of experts that specialize in each of these groups. The application of this rapidly advancing technology has the potential to provide a much more holistic, representative view of phytobenthic composition (Groendahl et al., 2017).

## CONFLICT OF INTEREST

None declared.

## AUTHOR CONTRIBUTIONS

FR, AB, and KT devised the concept and designed the study. FR, AB, KT, and VV carried out the sampling. VV prepared the samples for sequencing and the bioinformatic analyses for OTU clustering. KT carried out the statistical analyses and tests, index development, and wrote the manuscript together with the supporting material and figures. FR, AB, CSK, and JP provided constructive feedback and suggestions toward improving the manuscript.

## DATA ACCESSIBILITY

All data are uploaded as supporting material and also stored in Zenodo file repository (https://doi.org/10.5281/zenodo.1443416).
