# Supplementary figures and images for: The impact of OTU sequence similarity threshold on diatom‐based bioassessment: A case study of the rivers of Mayotte (France, Indian Ocean)
Source: Ecol Evol. 2018 Dec 18;9(1):166–79. doi: 10.1002/ece3.4701 (PMC6342121; doi:10.1002/ece3.4701)

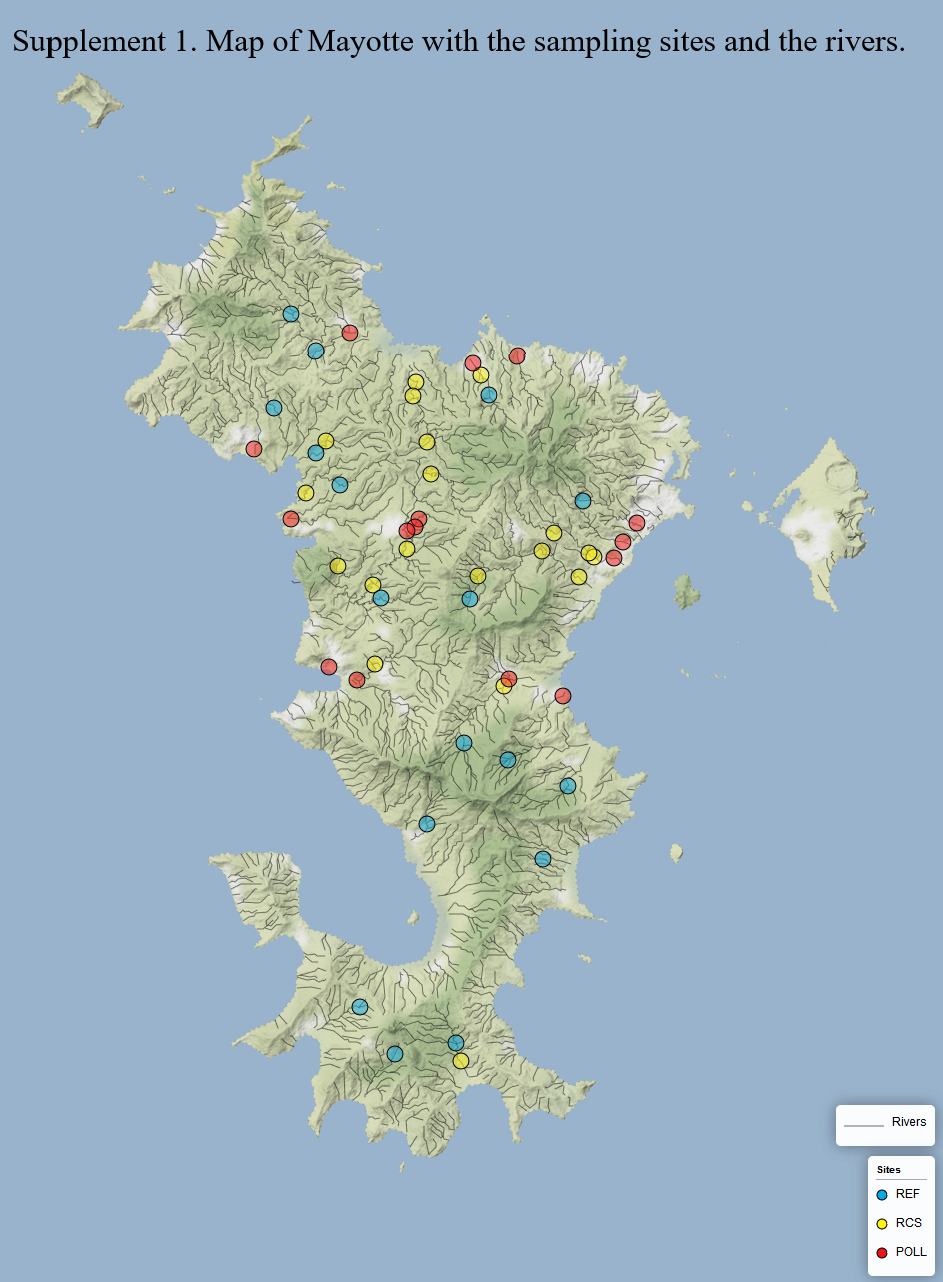

Supplement: Supplementary file 1 [file ECE3-9-166-s001.png]
